# Supplementary material for: Characterizing Relevant MicroRNA Editing Sites in Parkinson’s Disease
Source: Cells. 2022 Dec 24;12(1):75. doi: 10.3390/cells12010075 (PMC9818192; doi:10.3390/cells12010075)
Supplement: Supplementary file 1 [file cells-12-00075-s001.zip › SupplementaryFigures-2022-12-07.pdf]

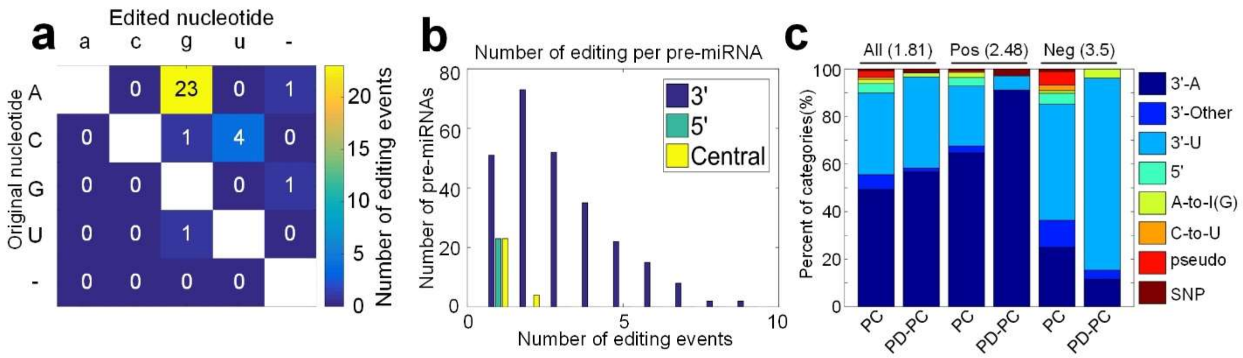

Supplementary Figure S1. The distribution of different types of editing sites in miRNAs. (**a**) The numbers of different types of editing events that do not happen at the 5' or 3' end of mature miRNAs. (**b**) The distribution of the numbers of pre-miRNAs with different numbers of 5'-, 3'- and Central editing sites, i.e. editing sites that do not happen at the ends of mature miRNAs. (**c**) The percentages of different types of miRNA editing sites whose editing levels are significantly correlated with the ages of individuals in PC and PD-PC samples, respectively. The values in the parenthesis are the Kullback-Leibler divergences between the distributions of PC and PD-PC. The source data are provided in Supplementary Table S12.

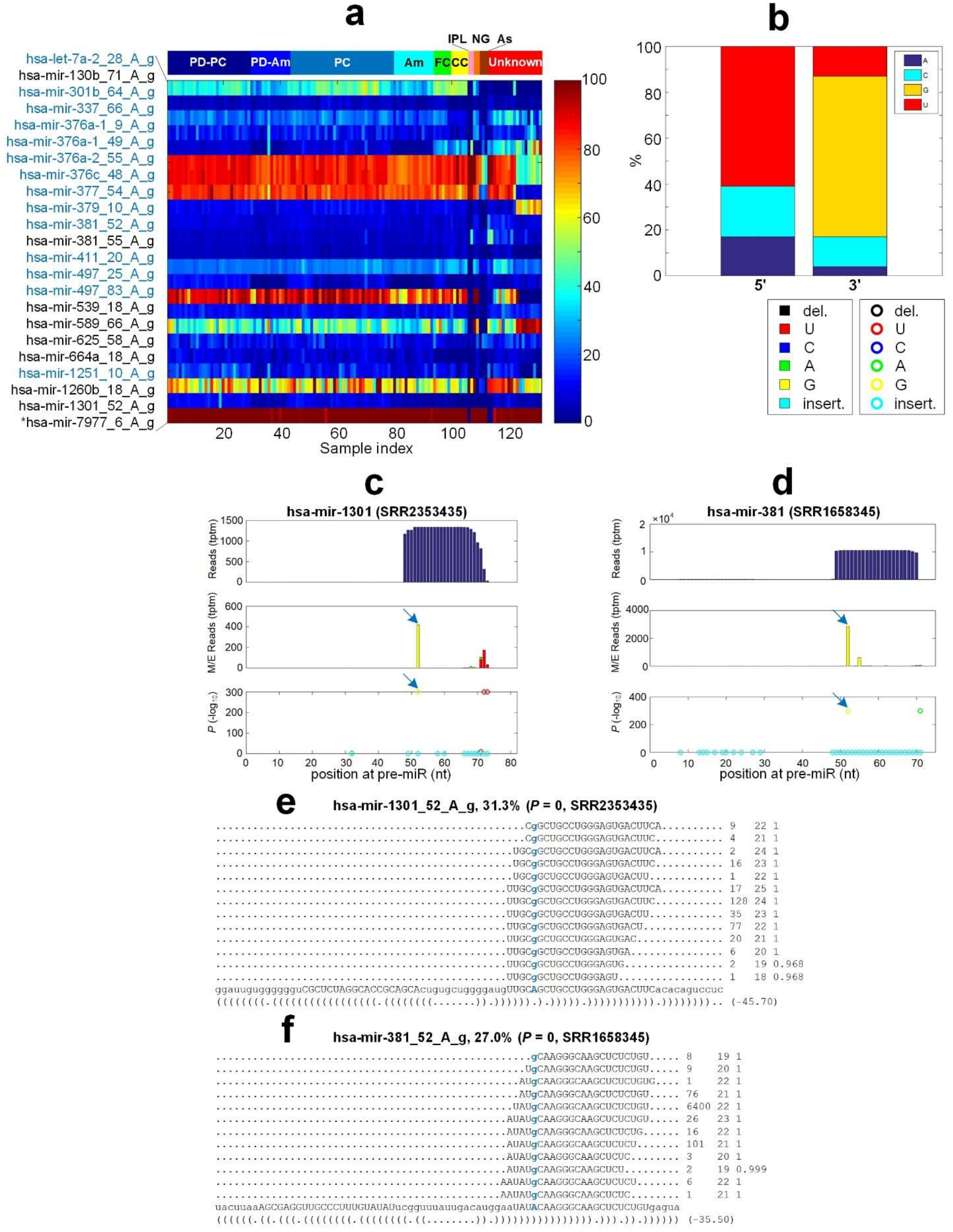
a
IPL NG As
PD-PC PD-Am PC Am FC CC Unknown
hsa-let-7a-2_28_A_g
hsa-mir-130b_71_A_g
hsa-mir-301b_64_A_g
hsa-mir-337_66_A_g
hsa-mir-376a-1_9_A_g
hsa-mir-376a-1_49_A_g
hsa-mir-376a-2_55_A_g
hsa-mir-376c_48_A_g
hsa-mir-377_54_A_g
hsa-mir-379_10_A_g
hsa-mir-381_52_A_g
hsa-mir-381_55_A_g
hsa-mir-411_20_A_g
hsa-mir-497_25_A_g
hsa-mir-497_83_A_g
hsa-mir-539_18_A_g
hsa-mir-589_66_A_g
hsa-mir-625_58_A_g
hsa-mir-664a_18_A_g
hsa-mir-1251_10_A_g
hsa-mir-1260b_18_A_g
hsa-mir-1301_52_A_g
*hsa-mir-7977_6_A_g
Sample index
b
%
5'
3'
A C G U
del. U C A G insert.
c
hsa-mir-1301 (SRR2353435)
d
hsa-mir-381 (SRR1658345)
Reads (tptm)
M/E Reads (tptm)
P (-log10)
position at pre-miR (nt)
e
hsa-mir-1301_52_A_g, 31.3% (P = 0, SRR2353435)
CgGCUGCCUGGGAGUGACUUCA 9 22 1
CgGCUGCCUGGGAGUGACUUC 4 21 1
UGCgGCUGCCUGGGAGUGACUUCA 2 24 1
UGCgGCUGCCUGGGAGUGACUUC 16 23 1
UGCgGCUGCCUGGGAGUGACUU 1 22 1
UUGCgGCUGCCUGGGAGUGACUUCA 17 25 1
UUGCgGCUGCCUGGGAGUGACUUC 128 24 1
UUGCgGCUGCCUGGGAGUGACUU 35 23 1
UUGCgGCUGCCUGGGAGUGACU 77 22 1
UUGCgGCUGCCUGGGAGUGAC 20 21 1
UUGCgGCUGCCUGGGAGUGA 6 20 1
UUGCgGCUGCCUGGGAGUG 2 19 0.968
UUGCgGCUGCCUGGGAGU 1 18 0.968
ggauugugggggguCGCUCUAGGCACCGCAGCAcugugcuggggaugUUGCAGCUGCCUGGGAGUGACUUCacacaguccuc
(-45.70)
f
hsa-mir-381_52_A_g, 27.0% (P = 0, SRR1658345)
gCAAGGGCAAGCUCUCUGU 8 19 1
UgCAAGGGCAAGCUCUCUGU 9 20 1
AUgCAAGGGCAAGCUCUCUGUG 1 22 1
AUgCAAGGGCAAGCUCUCUGU 76 21 1
UAUgCAAGGGCAAGCUCUCUGU 6400 22 1
AUAUgCAAGGGCAAGCUCUCUGU 26 23 1
AUAUgCAAGGGCAAGCUCUCUG 16 22 1
AUAUgCAAGGGCAAGCUCUCU 101 21 1
AUAUgCAAGGGCAAGCUCUC 3 20 1
AUAUgCAAGGGCAAGCUCU 2 19 0.999
AAUAUgCAAGGGCAAGCUCUCU 6 22 1
AAUAUgCAAGGGCAAGCUCUC 1 21 1
uacuuaaAGCGAGGUUGCCCUUUGUAUAUucgguuuauugacauggaaUAUACAAGGGCAAGCUCUCUGUgagua
(-35.50)

Supplementary Figure S2. The details of 23 identified A-to-I editing sites in miRNAs. (**a**) The editing levels of the 23 A-to-I editing sites in the 131 selected brain data sets. The 14 sites with blue names are conserved A-to-I editing sites in mammals. The 131 samples include 29 prefrontal cortex samples of postmortem PD patients (PD-PC), 14 amygdalae samples of postmortem PD patients (PD-Am), 36 prefrontal cortex samples of normal controls (PC), 14 amygdalae samples of normal controls (Am), 6 frontal cortex of normal controls (FC), 6 corpus callosum samples of normal controls (CC), 2 inferior parietal lobe samples of normal controls (IPL), 2 temporal neocortex gray matter samples of normal controls (NG), 3 astrocyte cell lines of normal controls (As), and 19 unknown brain regions of normal controls (Unknown). (**b**) The percentages of nucleotides beside the 23 A-to-I editing sites. (**c**) The MiRME map of hsa-mir-1301 in one of the PD-PC samples (SRR2353435). The upper panel shows the total numbers of reads that cover each nucleotide of the pre-miRNA. The central panel shows the numbers of M/E reads at each position of the pre-miRNA. And the lower panel givens multiple test corrected *P*-values (-log10 scaled) of the corresponding mutation/editing sites shown in the central panel. (**d**) The MiRME map of hsa-mir-381 in one of the unknown normal samples (SRR1658345). (**e**) The details of hsa-mir-1301_52_A_g in SRR2353435. (**f**) The details of hsa-mir-381_52_A_g in SRR1658345. In Part (e) and (f), the edited nucleotides are shown in bold face. The source data are provided in Supplementary Table S13.

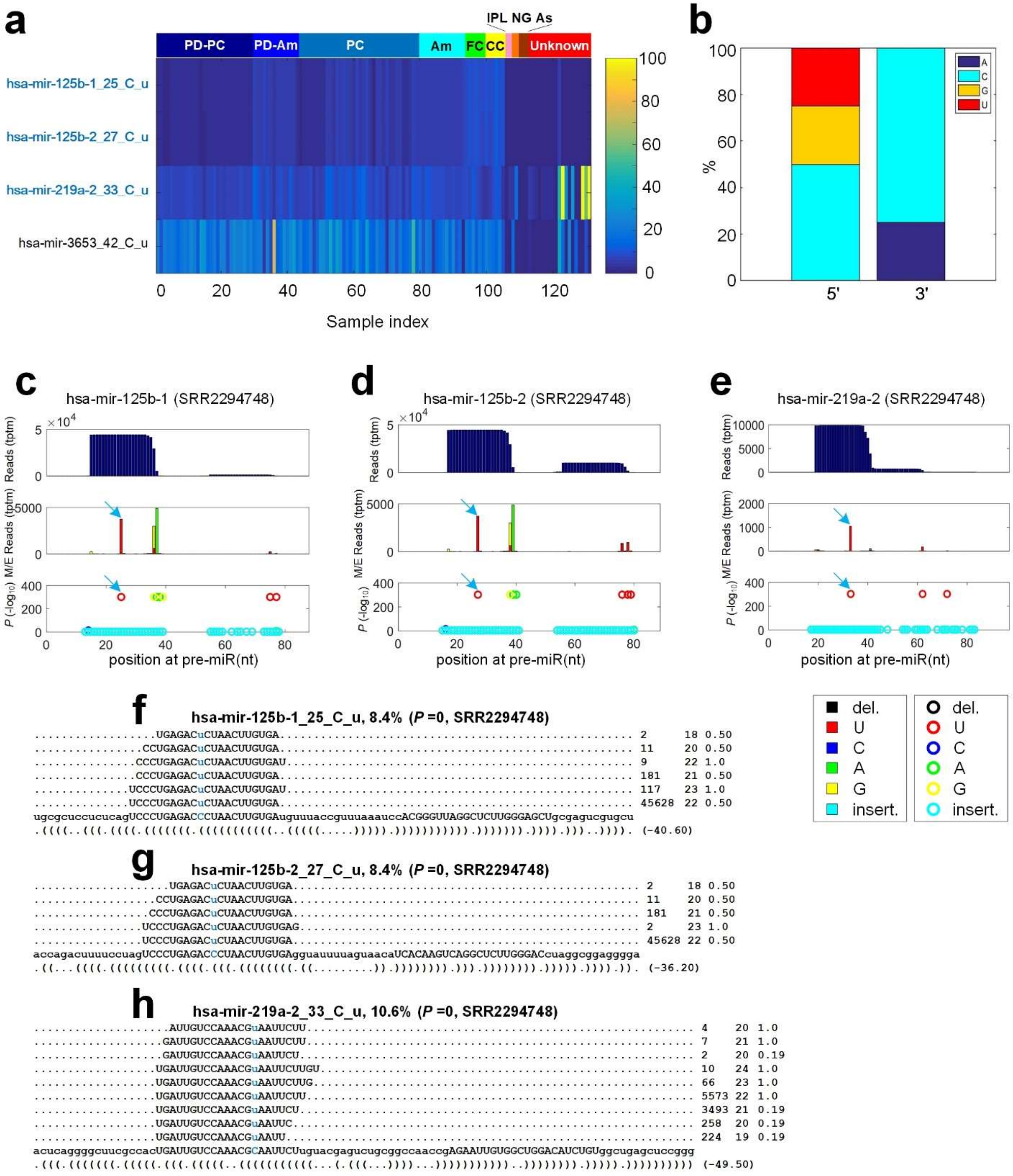

Supplementary Figure S3. The details of 4 identified C-to-U editing sites in miRNAs. (**a**) The editing levels of the 4 identified C-to-U editing sites in the 131 sRNA-seq profiles. The numbers of samples from different groups are the same as those in Figure S2a. The 3 sites with blue names are conserved C-to-U editing sites in primates. (**b**) The percentages of nucleotides beside these 4 C-to-U editing sites. (**c**) The MiRME map of hsa-mir-125b-1_25_C_u in one of the normal prefrontal cortex (PC) samples (SRR2294748). (**d**) The MiRME map of hsa-mir-125b-2

in one of the corpus callosum (CC) samples (SRR2294748). (**e**) The MiRME map of hsa-mir-219a-2 in one of corpus callosum (CC) samples (SRR2294748). (**f**) The details ofhsa-mir-125b-1_25_C_u in (SRR2294748). (**g**) The details of hsa-mir-125b-2_27_C_u in SRR2294748. (**h**) The details of hsa-mir-219a-2_72_C_u in (SRR2294748). In Part (f) to (h), the three numbers after the reads are the raw frequencies, length (in nt), and the weights of reads at the locus. The source data are provided in Supplementary Table S14.

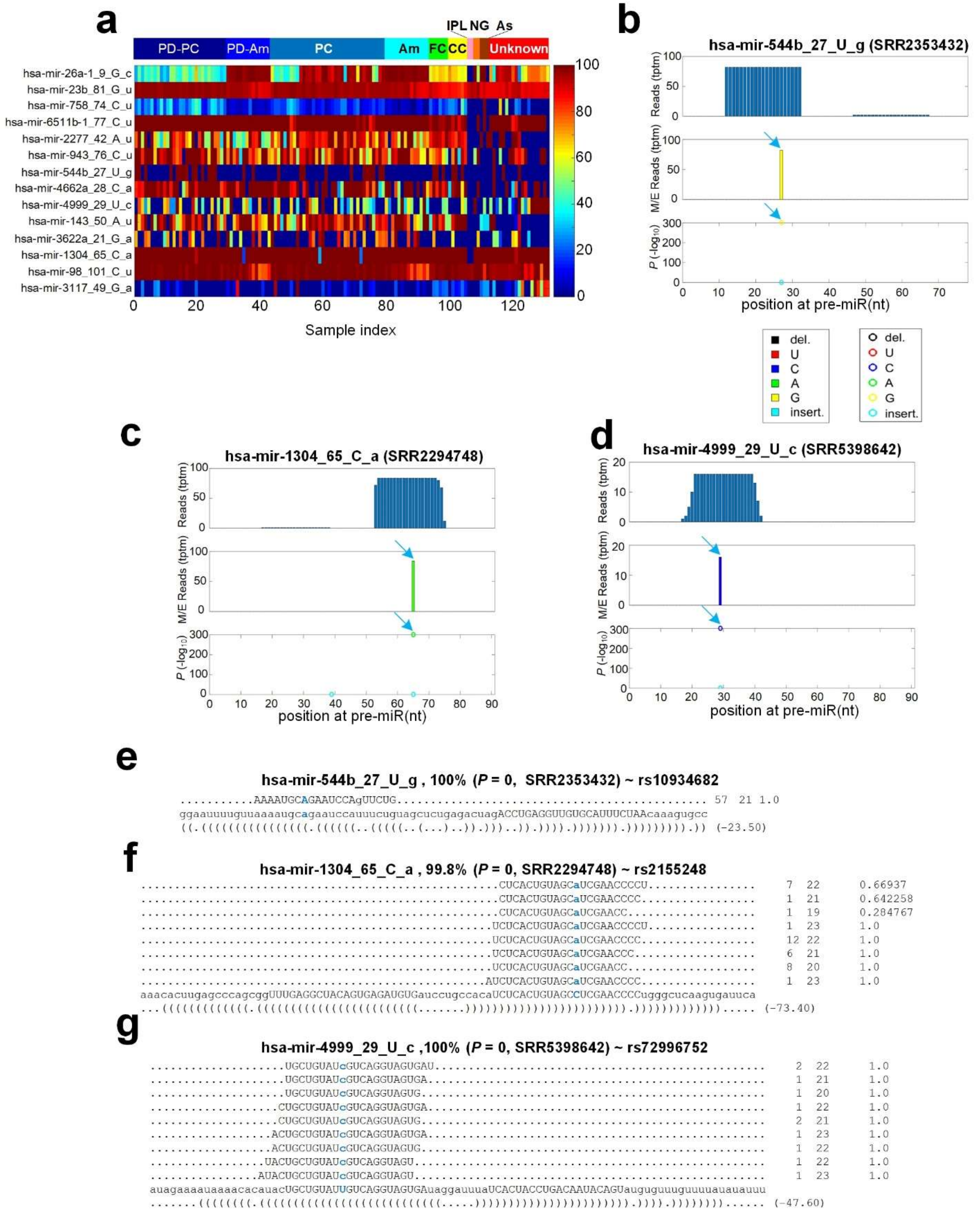

Supplementary Figure S4. The details of 14 identified SNPs in miRNAs. (**a**) The M/E levels of the 14 SNPs in the 131 sRNA-seq profiles. The numbers of samples from different groups are the same as those in Figure 2a. (**b**) The MiRME map of hsa-mir-544b in one of the prefrontal

cortex samples (SRR2353432). (**c**) The MiRME map of hsa-mir-1304 in one of the control corpus callosum samples (SRR2294748). (**d**) The MiRME map of hsa-mir-4999 in one the normal amygdala samples (SRR5398642). (**e**) The details of hsa-mir-544b_27_U_g, i.e., rs10934682, in SRR2353432. (**f**) The details of hsa-mir-1304_65_C_a, i.e., rs2155248, in SRR2294748. (**g**) The details of hsa-mir-4999_29_U_c, i.e., rs72996752, in SRR5398642. The source data are provided in Supplementary Table S15.

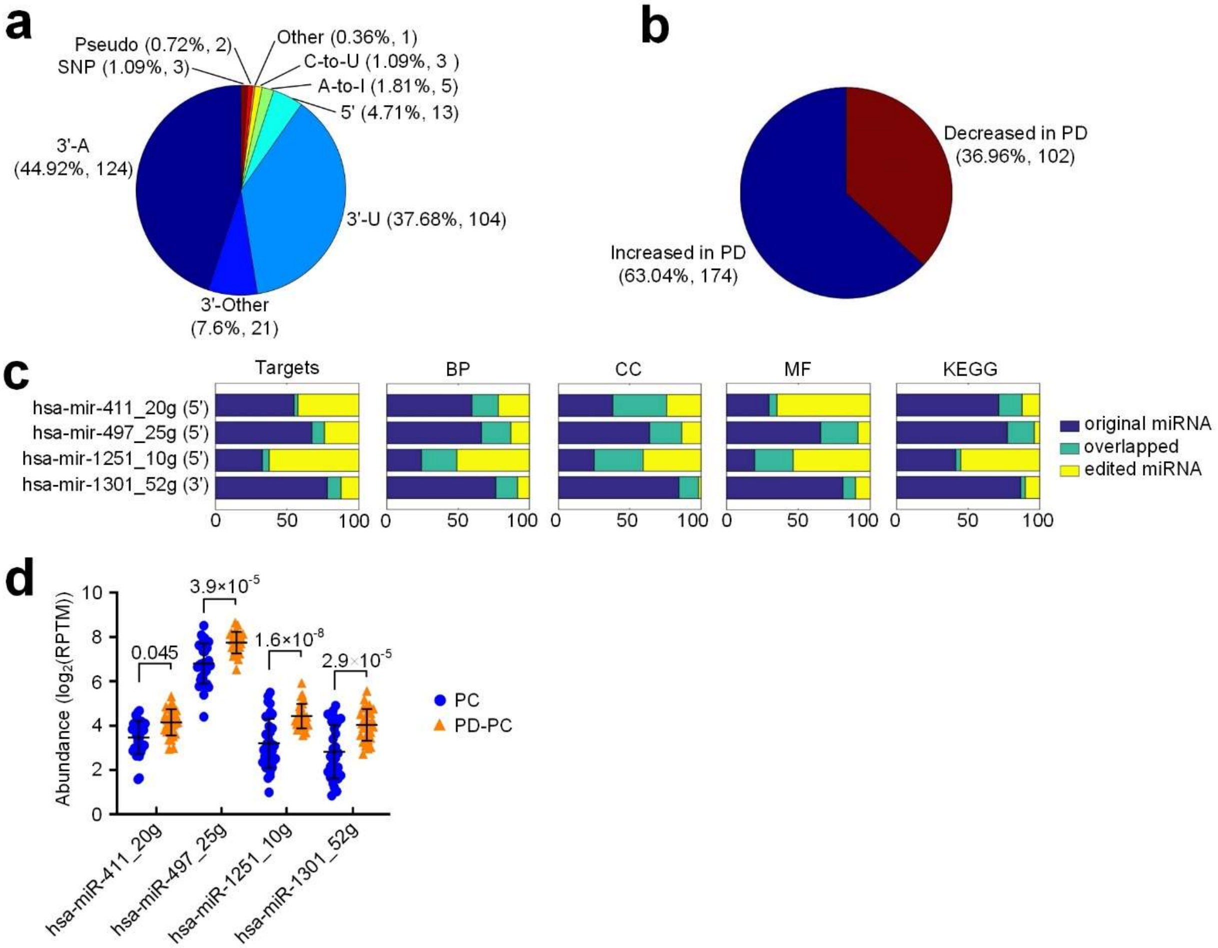

Supplementary Figure S5. The distribution of PD relevant editing sites. (**a**) The distribution of the different types of editing sites that have significantly different editing levels in PD-PC samples compared to the PC samples. (**b**) The distribution of sites in Part (a) whose editing levels are increased or decreased in PD-PC samples when compared to PC samples. (**c**) The comparisons of targets, GO terms, and KEGG pathways of original and four A-to-I edited miRNAs. The GO terms were shown in three major categories, i.e., Biological Process (BP), Cellular Component (CC) and Molecular Function (MF) separately. (**d**) The comparisons of normalized abundances of four A-to-I edited miRNAs in PC and PD-PC samples. The likelihood ratio tests (in edgeR) were used to compare the abundances of the edited miRNAs in different groups and to produce the *P*-values which were corrected with the Benjamini and Hochberg method. The multiple test corrected *P*-values were listed above the lines between PC and PD-PC samples. The source data are provided in Supplementary Table S16.

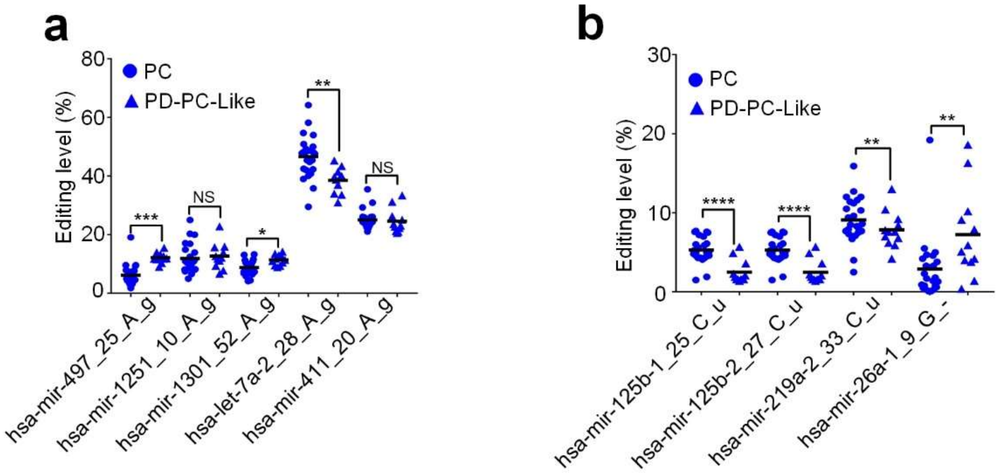

Supplementary Figure S6. The plot of the editing levels of five A-to-I and four other types of M/E sites in PC samples and PD-PC-like samples. (**a**) - (**b**) The compare of the editing levels of five A-to-I and four other types of M/E sites in PC and PD-PC-like samples. "*": Corrected $P< 0.05$, "**": Corrected $P < 0.01$, "***": Corrected $P < 0.001$, "****": Corrected $P < 0.0001$, and "NS": not significant, Mann-Whitney *U*-test. The source data are provided in Supplementary Table S17.

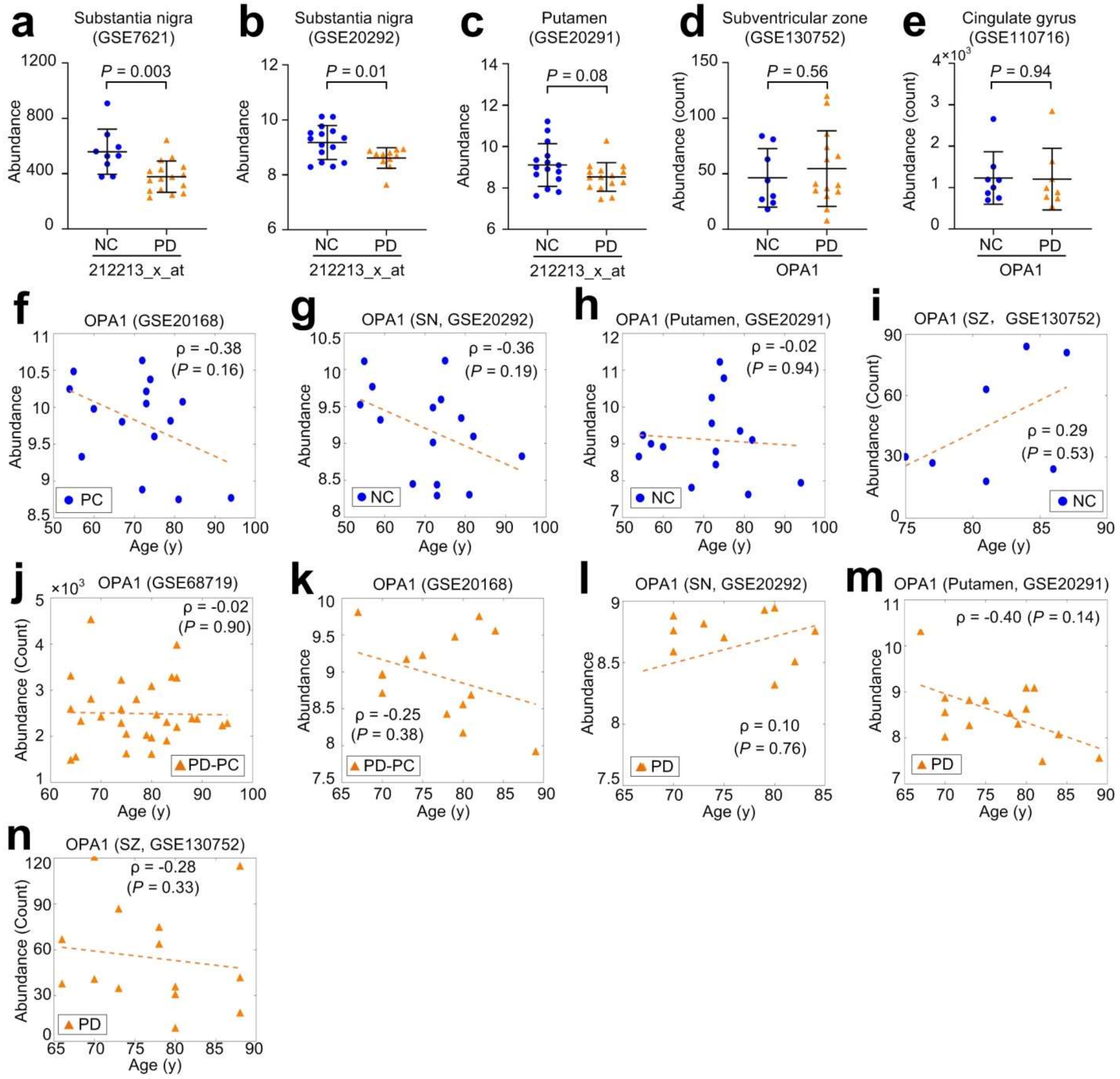

Supplementary Figure S7. The expression of OPA1 in different brain regions of PD patients and normal controls. (**a**) The abundance of OPA1 in substantia nigra samples of Normal controls (NC) and PD patients (PD) (GSE7621). The *P*-value was calculated with the two-tailed *t*-test. (**b**) The abundance of OPA1 in substantia nigra samples of normal controls (NC) and PD patients (PD) (GSE20292). The *P*-value was calculated with the two-tailed *t*-test. (**c**) The abundance of OPA1 in putamen samples of normal controls (NC) and PD patients (PD) (GSE20291). The *P*-value was calculated with the two-tailed *t*-test. (**d**) The abundance of OPA1 in subventricular zone samples of normal controls (NC) and PD patients (PD) (GSE130752). The *P*-value was calculated with the two-tailed *t*-test. (**e**) The abundance of OPA1 in cingulate gyrus samples of normal controls (NC) and PD patients (PD) (GSE110716). The *P*-value was calculated with the

two-tailed *t*-test. (**f**) The Spearman correlation (ρ) between the ages of PC samples and the abundances of OPA1 in GSE20168. (**g**) The Spearman correlation (ρ) between the ages of substantia nigra samples of normal controls (NC) and the abundances of OPA1 in GSE20292. (**h**) The Spearman correlation (ρ) between the ages of putamen samples of normal controls (NC) and the abundances of OPA1 in GSE20291. (**i**) The Spearman correlation between the ages of subventricular zone (SZ) samples of normal controls (NC) and the abundances of OPA1 in GSE130752. (**j**) The Spearman correlation (ρ) between the ages of PD-PC samples and the abundances of OPA1 in GSE68917. (**k**) The Spearman correlation (ρ) between the ages of PD-PC samples and the abundances of OPA1 in GSE20168. (**l**) The Spearman correlation between the ages of substantia nigra (SN) samples of PD patients (PD) and the abundances of OPA1 in GSE20292. (**m**) The Spearman correlation between the ages of the putamen samples of PD patients (PD) and the abundances of OPA1 in GSE20291. (**n**) The Spearman correlation between the ages of subventricular zone (SZ) samples of PD patients (PD) and the abundances of OPA1 in GSE130752. The source data are provided in Supplementary Table S18.

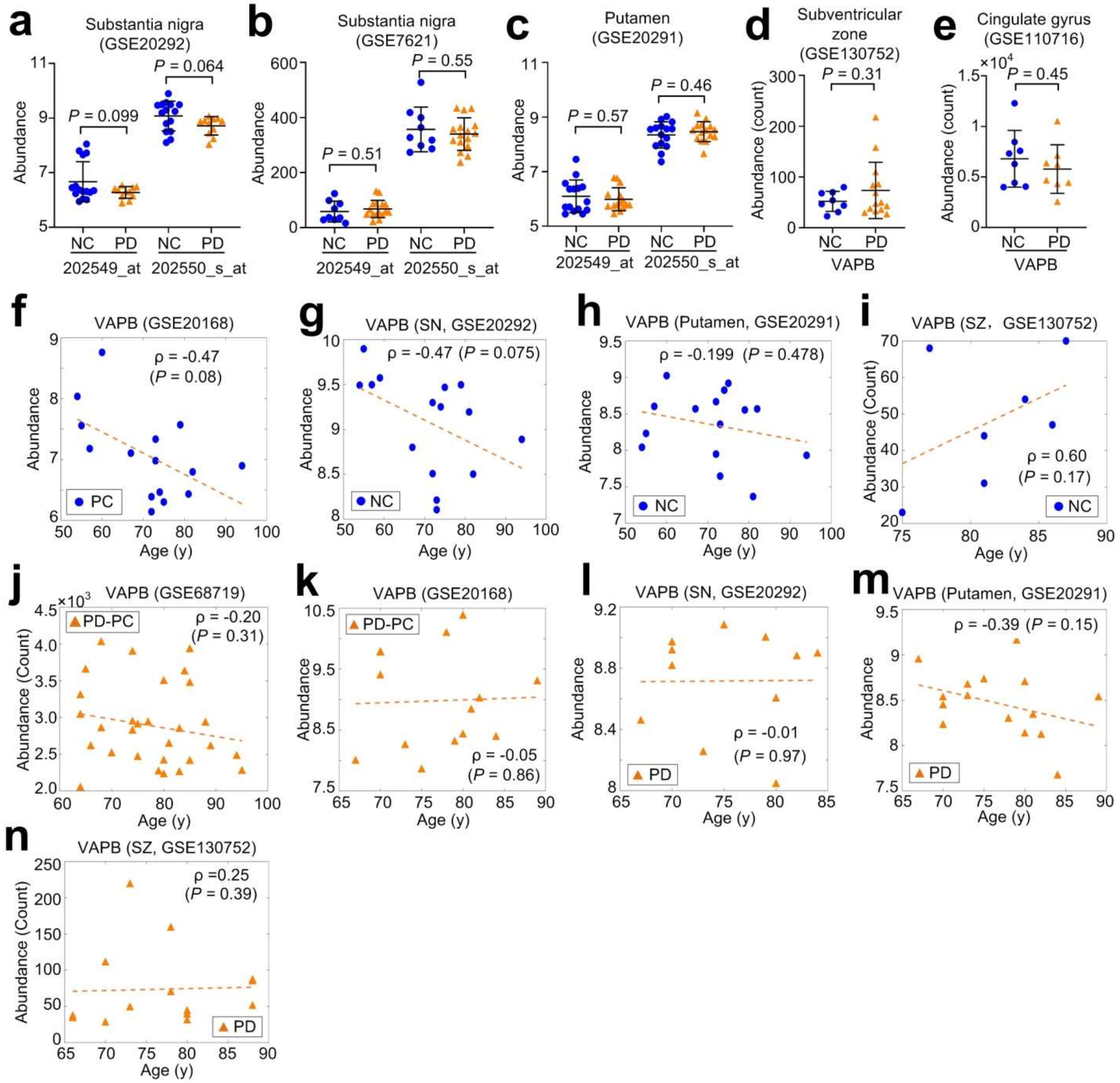

Supplementary Figure S8. The expression of VAPB in different brain regions of PD patients and normal controls. (**a**) The abundance of VAPB in substantia nigra samples of Normal controls (NC) and PD patients (PD) (GSE20292). The *P*-value was calculated with the two-tailed *t*-test. (**b**) The abundance of VAPB in substantia nigra samples of PD patients (PD) and normal controls (NC) (GSE7621). The *P*-value was calculated with the two-tailed *t*-test. (**c**) The abundance of VAPB in putamen samples of Normal controls (NC) and PD patients (PD) (GSE20291). The *P*-value was calculated with the two-tailed *t*-test. (**d**) The abundance of VAPB in subventricular zone samples of PD patients (PD) and normal controls (NC) (GSE130752). The *P*-value was calculated with the two-tailed *t*-test. (**e**) The abundance of VAPB in cingulate gyrus samples of PD patients (PD) and normal controls (NC) (GSE110716). The *P*-value was calculated with the two-tailed *t*-test. (**f**) The Spearman correlation (ρ) between the ages of PC

samples and the abundances of VAPB in GSE20168. (**g**) The Spearman correlation (ρ) between the ages of substantia nigra samples of normal controls (NC) and the abundances of VAPB in GSE20292. (**h**) The Spearman correlation (ρ) between the ages of putamen samples of normal controls (NC) and the abundances of VAPB in GSE20291. (**i**) The Spearman correlation between the ages of subventricular zone (SZ) samples of normal controls (NC) and the abundances of VAPB in GSE130752. (**j**) The Spearman correlation (ρ) between the ages of PD-PC samples and the abundances of VAPB in GSE68917. (**k**) The Spearman correlation (ρ) between the ages of PD-PC samples and the abundances of VAPB in GSE20168. (**l**) The Spearman correlation between the ages of substantia nigra (SN) samples of PD patients (PD) and the abundances of VAPB in GSE20292. (**m**) The Spearman correlation between the ages of the putamen samples of PD patients (PD) and the abundances of VAPB in GSE20291. (**n**) The Spearman correlation between the ages of subventricular zone (SZ) samples of PD patients (PD) and the abundances of VAPB in GSE130752. The source data are provided in Supplementary Table S19.

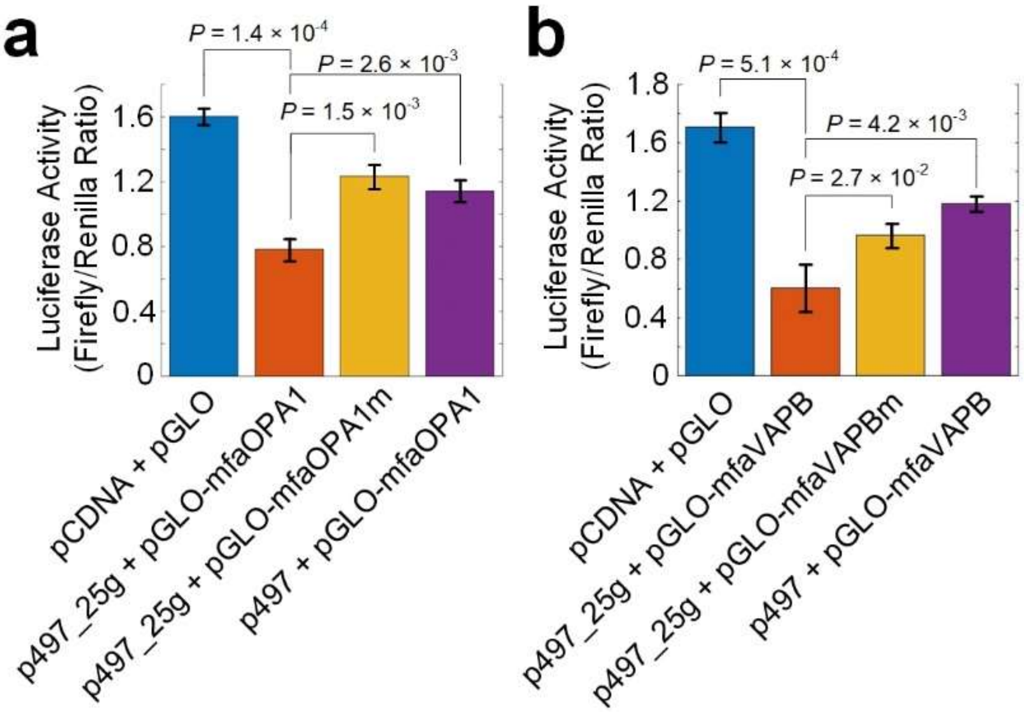

Supplementary Figure S9. Validation of monkey OPA1 and VAPB as targets of A-to-I edited miR-497-5p. (**a**) The luciferase activities when co-transfecting a pGLO plasmid of 3' UTRs of monkey OPA1 in Part (a) of Figure 5 and a pCDNA plasmid containing original pre-hsa-mir-497 (p497) or pre-hsa-mir-497_25g (p497_25g), respectively. (**b**) The luciferase activities when co-transfecting a pGLO plasmid of 3' UTRs of monkey VAPB in Part (a) of Figure 5 and a pCDNA plasmid containing original pre-hsa-mir-497 (p497) or pre-hsa-mir-497_25g (p497_25g), respectively. In Part (a) to (b), the values shown are mean values ± SDs. *P*-values were based on two-tailed *t*-tests. The source data are available in Supplementary Table S20.
